# Crystal structure of 1-benzoyl-3-(4-fluoro­phen­yl)thio­urea

**DOI:** 10.1107/S1600536814018376

**Published:** 2014-08-16

**Authors:** Rabab Sharaf Jassas, Abdullah M. Asiri, Muhammad Nadeem Arshad, Mohie E. M. Zayed, Ghulam Mustafa

**Affiliations:** a Chemistry Department, Faculty of Science, King Abdulaziz University, PO Box 80203, Jeddah 21589, Saudi Arabia; b Center of Excellence for Advanced Materials Research (CEAMR), King Abdulaziz University, PO Box 80203, Jeddah 21589, Saudi Arabia; c Department of Chemistry, University of Gujrat, Gujrat 50700, Pakistan

**Keywords:** crystal structure, thio­urea, amide, hydrogen-bonded dimers

## Abstract

The title compound, C_14_H_11_FN_2_OS, contains two mol­ecules (*A* and *B*) in the asymmetric unit, with different conformations. In mol­ecule *A*, the dihedral angles between the central thio­urea grouping and the phenyl and fluoro­benzene rings are 28.77 (8) and 41.82 (8)°, respectively, and the dihedral angle between the ring planes is 70.02 (9)°. Equivalent data for mol­ecule *B* are 8.46 (8), 47.78 (8) and 52.99 (9)°, respectively. Both mol­ecules feature an intra­molecular N—H⋯O hydrogen bond, which closes an *S*(6) ring. In the crystal, *A*+*B* dimers linked by pairs of N—H⋯S hydrogen bonds generate *R*_2_^2^(8) loops.

## Related literature   

For related structures, see: Othman *et al.* (2010 ▶); Rauf *et al.* (2012 ▶) Saeed & Flörke (2006*a* ▶, 2006*b* ▶); Saeed *et al.* (2011 ▶); Yamin & Yusof (2003*a* ▶,*b* ▶).
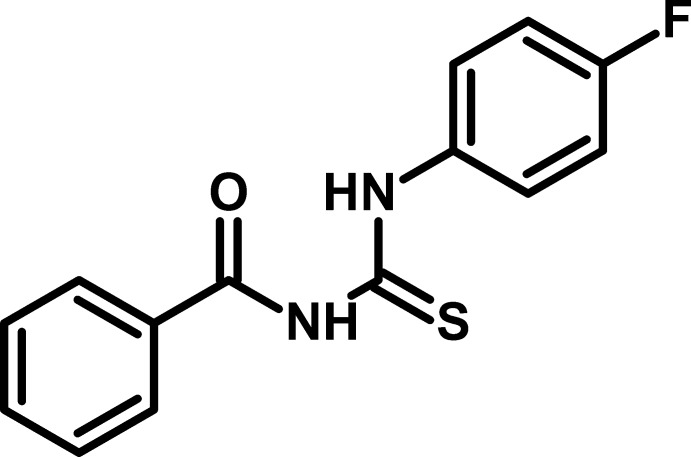


## Experimental   

### Crystal data  

C_14_H_11_FN_2_OS
*M* *_r_* = 274.31
Triclinic, 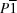

*a* = 9.6265 (4) Å
*b* = 11.1329 (4) Å
*c* = 13.8252 (5) Å
α = 110.646 (3)°
β = 100.708 (3)°
γ = 102.762 (3)°
*V* = 1294.58 (9) Å^3^
*Z* = 4
Cu *K*α radiation
μ = 2.28 mm^−1^
*T* = 296 K
0.36 × 0.28 × 0.22 mm

### Data collection  

Agilent SuperNova, Dual, Cu at zero, Atlas, CCD diffractometer
Absorption correction: multi-scan (*CrysAlis PRO*; Agilent, 2012 ▶) *T*_min_ = 0.817, *T*_max_ = 1.000
11636 measured reflections
5354 independent reflections
4757 reflections with *I* > 2σ(*I*)
*R*_int_ = 0.015

### Refinement  

*R*[*F* ^2^ > 2σ(*F* ^2^)] = 0.036
*wR*(*F* ^2^) = 0.102
*S* = 1.03
5354 reflections
346 parameters
H atoms treated by a mixture of independent and constrained refinement
Δρ_max_ = 0.20 e Å^−3^
Δρ_min_ = −0.35 e Å^−3^

### 

Data collection: *CrysAlis PRO* (Agilent, 2012 ▶); cell refinement: *CrysAlis PRO*; data reduction: *CrysAlis PRO*; program(s) used to solve structure: *SHELXS97* (Sheldrick, 2008 ▶); program(s) used to refine structure: *SHELXL97* (Sheldrick, 2008 ▶); molecular graphics: *PLATON* (Spek, 2009 ▶); software used to prepare material for publication: *WinGX* (Farrugia, 2012 ▶) and *X-SEED* (Barbour, 2001 ▶).

## Supplementary Material

Crystal structure: contains datablock(s) I, New_Global_Publ_Block. DOI: 10.1107/S1600536814018376/hb7271sup1.cif

Structure factors: contains datablock(s) I. DOI: 10.1107/S1600536814018376/hb7271Isup2.hkl

Supporting information file. DOI: 10.1107/S1600536814018376/hb7271Isup3.cml

**Fig. 1.**
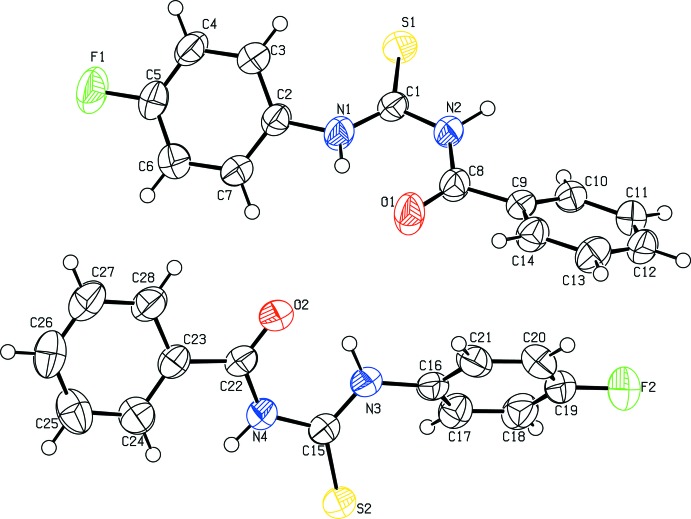
The molecular structure of (I) with 50% displacement ellipsoids.

. DOI: 10.1107/S1600536814018376/hb7271fig1.tif
The mol­ecular structure of (I) with 50% displacement ellipsoids.

**Fig. 2.**
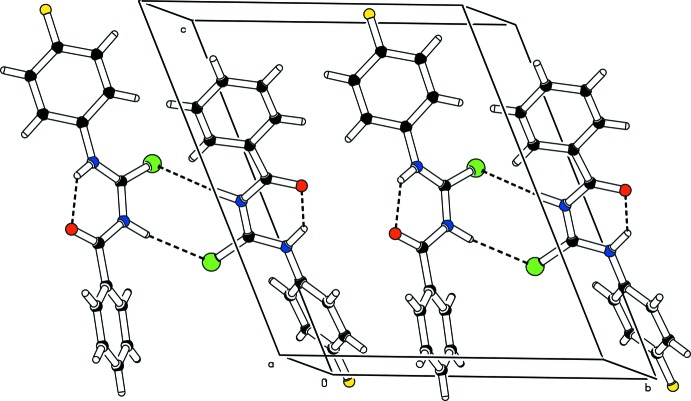
The inter and intramolecular hydrogen bonding shown using dashed lines.

. DOI: 10.1107/S1600536814018376/hb7271fig2.tif
The inter and intra­molecular hydrogen bonding shown using dashed lines.

CCDC reference: 1018979

Additional supporting information:  crystallographic information; 3D view; checkCIF report

## Figures and Tables

**Table 1:** Hydrogen-bond geometry (Å, °)

*D*—H⋯*A*	*D*—H	H⋯*A*	*D*⋯*A*	*D*—H⋯*A*
N2—H2⋯S2^i^	0.99	2.56	3.5433 (13)	170
N4—H4⋯S1^i^	0.93	2.74	3.5976 (13)	154
N1—H1⋯O1	0.86 (2)	1.95 (2)	2.6408 (17)	137.5 (19)
N3—H3⋯O2	0.98	1.81	2.6307 (17)	139

Symmetry code: (i) 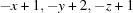.

## supplementary crystallographic information

### S1. Synthesis and crystallization

Benzoyl­iso­thio­cyanate (1 g, 6.13 mmol) was dissolved in acetone (50 ml) and allowed to stirr for an hour. Then 4-flouroaniline(0.68 g, 6.13 mmol) was added to the above mixture and stirred for another 1 h. The completion of reaction was checked by thin layer chromatography (TLC). The mixture was poured into acidified water under stirring. The precipitate obtained were separated and washed with deionized cold distilled water and recrystallization from ethanol solution under slow evaporation method to produce colorless blocks of title compound.

### S2. Refinement

All the aromatic C—H H-atoms were positioned with idealized geometry with C—H = 0.93 Å,for aromatic and refined as riding with *U*_iso_(H) = 1.2*U*_eq_(C). The N—H H-atoms were positioned in difference map and refined freely with N—H = 0.97 (4) Å, *U*_iso_(H) = 1.2 for N atoms.

### Crystal data

**Table:**

C_14_H_11_FN_2_OS	*V* = 1294.58 (9) Å^3^
*M**_r_* = 274.31	*Z* = 4
Triclinic, *P*1	*F*(000) = 568
Hall symbol: -P 1	*D*_x_ = 1.407 Mg m^−^^3^
*a* = 9.6265 (4) Å	Cu *K*α radiation, λ = 1.54184 Å
*b* = 11.1329 (4) Å	µ = 2.28 mm^−^^1^
*c* = 13.8252 (5) Å	*T* = 296 K
α = 110.646 (3)°	Block, colorless
β = 100.708 (3)°	0.36 × 0.28 × 0.22 mm
γ = 102.762 (3)°	

### Data collection

**Table:**

Agilent SuperNova, Dual, Cu at zero, Atlas, CCD diffractometer	5354 independent reflections
Radiation source: SuperNova (Cu) X-ray Source	4757 reflections with *I* > 2σ(*I*)
Mirror monochromator	*R*_int_ = 0.015
ω scans	θ_max_ = 76.5°, θ_min_ = 4.4°
Absorption correction: multi-scan (*CrysAlis PRO*; Agilent, 2012)	*h* = −12→11
*T*_min_ = 0.817, *T*_max_ = 1.000	*k* = −13→12
11636 measured reflections	*l* = −11→17

### Refinement

**Table:**

Refinement on *F*^2^	Primary atom site location: structure-invariant direct methods
Least-squares matrix: full	Secondary atom site location: difference Fourier map
*R*[*F*^2^ > 2σ(*F*^2^)] = 0.036	Hydrogen site location: inferred from neighbouring sites
*wR*(*F*^2^) = 0.102	H atoms treated by a mixture of independent and constrained refinement
*S* = 1.03	*w* = 1/[σ^2^(*F*_o_^2^) + (0.0538*P*)^2^ + 0.268*P*] where *P* = (*F*_o_^2^ + 2*F*_c_^2^)/3
5354 reflections	(Δ/σ)_max_ = 0.001
346 parameters	Δρ_max_ = 0.20 e Å^−^^3^
0 restraints	Δρ_min_ = −0.35 e Å^−^^3^

### Special details

**Table:**

Geometry. All esds (except the esd in the dihedral angle between two l.s. planes) are estimated using the full covariance matrix. The cell esds are taken into account individually in the estimation of esds in distances, angles and torsion angles; correlations between esds in cell parameters are only used when they are defined by crystal symmetry. An approximate (isotropic) treatment of cell esds is used for estimating esds involving l.s. planes.
Refinement. Refinement of F^2^ against ALL reflections. The weighted R-factor wR and goodness of fit S are based on F^2^, conventional R-factors R are based on F, with F set to zero for negative F^2^. The threshold expression of F^2^ > 2sigma(F^2^) is used only for calculating R-factors(gt) etc. and is not relevant to the choice of reflections for refinement. R-factors based on F^2^ are statistically about twice as large as those based on F, and R- factors based on ALL data will be even larger.

### Fractional atomic coordinates and isotropic or equivalent isotropic displacement parameters (Å^2^)

**Table:**

	*x*	*y*	*z*	*U*_iso_*/*U*_eq_	
S1	0.10971 (5)	0.71506 (4)	0.58605 (3)	0.05533 (12)	
S2	0.99550 (5)	1.23786 (4)	0.68544 (4)	0.06368 (14)	
F1	0.42593 (15)	0.61882 (14)	1.00154 (8)	0.0807 (3)	
F2	1.30145 (16)	1.00061 (14)	1.00287 (10)	0.0960 (4)	
O1	0.30327 (17)	0.41067 (15)	0.38650 (9)	0.0768 (4)	
O2	0.67259 (14)	0.81808 (11)	0.48289 (10)	0.0626 (3)	
N1	0.28430 (16)	0.55698 (14)	0.57813 (10)	0.0533 (3)	
N2	0.17580 (15)	0.56414 (13)	0.41761 (9)	0.0483 (3)	
N3	0.91440 (14)	0.97471 (12)	0.64274 (10)	0.0513 (3)	
N4	0.78454 (14)	1.03902 (13)	0.52148 (10)	0.0504 (3)	
C1	0.19583 (17)	0.60743 (14)	0.52881 (11)	0.0454 (3)	
C2	0.31803 (16)	0.57760 (15)	0.68871 (11)	0.0456 (3)	
C3	0.3556 (2)	0.70415 (16)	0.77249 (13)	0.0559 (4)	
H3A	0.3568	0.7797	0.7578	0.067*	
C4	0.39115 (19)	0.71680 (17)	0.87823 (12)	0.0586 (4)	
H4A	0.4153	0.8006	0.9354	0.070*	
C5	0.39026 (18)	0.60464 (18)	0.89724 (12)	0.0543 (4)	
C6	0.3546 (2)	0.47939 (18)	0.81661 (14)	0.0583 (4)	
H6	0.3548	0.4046	0.8321	0.070*	
C7	0.31813 (18)	0.46637 (16)	0.71082 (12)	0.0526 (3)	
H7	0.2935	0.3819	0.6544	0.063*	
C8	0.22178 (18)	0.46495 (16)	0.35187 (12)	0.0505 (3)	
C9	0.16680 (16)	0.42877 (14)	0.23398 (11)	0.0438 (3)	
C10	0.02856 (17)	0.43219 (15)	0.18570 (12)	0.0475 (3)	
H10	−0.0331	0.4603	0.2274	0.057*	
C11	−0.01708 (19)	0.39336 (17)	0.07491 (13)	0.0549 (4)	
H11	−0.1100	0.3949	0.0423	0.066*	
C12	0.0745 (2)	0.35248 (17)	0.01270 (12)	0.0571 (4)	
H12	0.0438	0.3275	−0.0614	0.069*	
C13	0.2114 (2)	0.34884 (18)	0.06075 (13)	0.0585 (4)	
H13	0.2733	0.3216	0.0189	0.070*	
C14	0.25746 (18)	0.38540 (16)	0.17091 (12)	0.0524 (3)	
H14	0.3491	0.3809	0.2027	0.063*	
C15	0.89789 (16)	1.07566 (15)	0.61595 (12)	0.0466 (3)	
C16	1.01650 (17)	0.98710 (14)	0.73730 (12)	0.0470 (3)	
C17	1.16625 (18)	1.05699 (17)	0.76648 (13)	0.0541 (4)	
H17	1.2019	1.1011	0.7259	0.065*	
C18	1.2625 (2)	1.06121 (19)	0.85570 (15)	0.0637 (4)	
H18	1.3633	1.1082	0.8761	0.076*	
C19	1.2071 (2)	0.99480 (18)	0.91389 (14)	0.0635 (4)	
C20	1.0604 (2)	0.92345 (18)	0.88583 (16)	0.0668 (5)	
H20	1.0258	0.8785	0.9262	0.080*	
C21	0.9641 (2)	0.91938 (16)	0.79610 (15)	0.0584 (4)	
H21	0.8638	0.8708	0.7754	0.070*	
C22	0.67355 (17)	0.91742 (15)	0.46324 (12)	0.0482 (3)	
C23	0.55378 (16)	0.91245 (15)	0.37529 (12)	0.0475 (3)	
C24	0.53131 (18)	1.02572 (19)	0.36376 (15)	0.0609 (4)	
H24	0.5928	1.1114	0.4126	0.073*	
C25	0.4163 (2)	1.0108 (2)	0.27875 (18)	0.0735 (5)	
H25	0.4017	1.0869	0.2705	0.088*	
C26	0.3241 (2)	0.8846 (2)	0.20673 (16)	0.0721 (5)	
H26	0.2483	0.8755	0.1495	0.086*	
C27	0.3438 (2)	0.7719 (2)	0.21907 (15)	0.0713 (5)	
H27	0.2803	0.6866	0.1709	0.086*	
C28	0.45776 (19)	0.78475 (18)	0.30293 (14)	0.0610 (4)	
H28	0.4705	0.7082	0.3112	0.073*	
H2	0.1172	0.6095	0.3842	0.073*	
H4	0.7819	1.1067	0.4974	0.073*	
H3	0.8390	0.8878	0.5964	0.073*	
H1	0.316 (2)	0.497 (2)	0.5386 (17)	0.073*	

### Atomic displacement parameters (Å^2^)

**Table:**

	*U*^11^	*U*^22^	*U*^33^	*U*^12^	*U*^13^	*U*^23^
S1	0.0705 (3)	0.0524 (2)	0.0459 (2)	0.02927 (18)	0.02121 (17)	0.01513 (16)
S2	0.0710 (3)	0.0427 (2)	0.0561 (2)	0.00407 (18)	−0.00598 (19)	0.01671 (17)
F1	0.0990 (8)	0.1050 (9)	0.0398 (5)	0.0368 (7)	0.0174 (5)	0.0300 (5)
F2	0.1099 (10)	0.0882 (9)	0.0716 (7)	0.0323 (7)	−0.0158 (7)	0.0321 (7)
O1	0.1027 (10)	0.0990 (10)	0.0416 (6)	0.0699 (9)	0.0178 (6)	0.0220 (6)
O2	0.0697 (7)	0.0427 (6)	0.0563 (7)	0.0099 (5)	−0.0017 (5)	0.0133 (5)
N1	0.0669 (8)	0.0583 (8)	0.0355 (6)	0.0310 (7)	0.0137 (6)	0.0137 (5)
N2	0.0616 (7)	0.0484 (7)	0.0353 (6)	0.0238 (6)	0.0130 (5)	0.0140 (5)
N3	0.0537 (7)	0.0411 (6)	0.0465 (7)	0.0127 (5)	0.0024 (5)	0.0112 (5)
N4	0.0534 (7)	0.0444 (6)	0.0437 (6)	0.0091 (5)	0.0043 (5)	0.0158 (5)
C1	0.0529 (8)	0.0406 (7)	0.0372 (7)	0.0126 (6)	0.0117 (6)	0.0118 (5)
C2	0.0477 (7)	0.0486 (8)	0.0357 (7)	0.0153 (6)	0.0101 (6)	0.0125 (6)
C3	0.0672 (10)	0.0456 (8)	0.0446 (8)	0.0137 (7)	0.0086 (7)	0.0131 (6)
C4	0.0646 (10)	0.0559 (9)	0.0372 (7)	0.0166 (7)	0.0060 (7)	0.0045 (6)
C5	0.0535 (8)	0.0705 (10)	0.0363 (7)	0.0190 (7)	0.0121 (6)	0.0194 (7)
C6	0.0665 (10)	0.0565 (9)	0.0523 (9)	0.0174 (8)	0.0129 (7)	0.0260 (7)
C7	0.0610 (9)	0.0454 (8)	0.0419 (7)	0.0172 (7)	0.0091 (6)	0.0098 (6)
C8	0.0583 (9)	0.0552 (8)	0.0385 (7)	0.0255 (7)	0.0136 (6)	0.0152 (6)
C9	0.0513 (7)	0.0404 (7)	0.0373 (7)	0.0153 (6)	0.0124 (6)	0.0130 (5)
C10	0.0515 (8)	0.0466 (7)	0.0440 (7)	0.0179 (6)	0.0142 (6)	0.0163 (6)
C11	0.0580 (9)	0.0562 (9)	0.0472 (8)	0.0172 (7)	0.0067 (7)	0.0220 (7)
C12	0.0724 (10)	0.0552 (9)	0.0378 (7)	0.0145 (8)	0.0122 (7)	0.0174 (7)
C13	0.0662 (10)	0.0641 (10)	0.0443 (8)	0.0213 (8)	0.0246 (7)	0.0162 (7)
C14	0.0520 (8)	0.0582 (9)	0.0449 (8)	0.0206 (7)	0.0148 (6)	0.0164 (7)
C15	0.0488 (7)	0.0439 (7)	0.0411 (7)	0.0128 (6)	0.0100 (6)	0.0130 (6)
C16	0.0516 (8)	0.0388 (7)	0.0444 (7)	0.0181 (6)	0.0095 (6)	0.0100 (6)
C17	0.0512 (8)	0.0591 (9)	0.0493 (8)	0.0194 (7)	0.0151 (7)	0.0177 (7)
C18	0.0534 (9)	0.0623 (10)	0.0594 (10)	0.0188 (8)	0.0046 (7)	0.0125 (8)
C19	0.0767 (11)	0.0530 (9)	0.0506 (9)	0.0284 (8)	−0.0005 (8)	0.0143 (7)
C20	0.0863 (13)	0.0541 (9)	0.0666 (11)	0.0264 (9)	0.0169 (9)	0.0322 (8)
C21	0.0587 (9)	0.0454 (8)	0.0697 (10)	0.0152 (7)	0.0107 (8)	0.0268 (7)
C22	0.0510 (8)	0.0445 (7)	0.0404 (7)	0.0131 (6)	0.0115 (6)	0.0096 (6)
C23	0.0444 (7)	0.0531 (8)	0.0406 (7)	0.0131 (6)	0.0142 (6)	0.0143 (6)
C24	0.0480 (8)	0.0605 (10)	0.0698 (11)	0.0093 (7)	0.0105 (7)	0.0298 (8)
C25	0.0551 (10)	0.0889 (14)	0.0904 (14)	0.0205 (9)	0.0156 (9)	0.0564 (12)
C26	0.0524 (9)	0.1022 (15)	0.0569 (10)	0.0162 (10)	0.0074 (8)	0.0364 (10)
C27	0.0607 (10)	0.0747 (12)	0.0524 (10)	0.0125 (9)	0.0015 (8)	0.0093 (9)
C28	0.0578 (9)	0.0569 (9)	0.0507 (9)	0.0143 (7)	0.0055 (7)	0.0093 (7)

### Geometric parameters (Å, º)

**Table:**

S1—C1	1.6623 (15)	C10—C11	1.388 (2)
S2—C15	1.6608 (15)	C10—H10	0.9300
F1—C5	1.3616 (17)	C11—C12	1.381 (2)
F2—C19	1.3585 (19)	C11—H11	0.9300
O1—C8	1.2181 (19)	C12—C13	1.377 (2)
O2—C22	1.2269 (19)	C12—H12	0.9300
N1—C1	1.3309 (19)	C13—C14	1.383 (2)
N1—C2	1.4250 (18)	C13—H13	0.9300
N1—H1	0.86 (2)	C14—H14	0.9300
N2—C8	1.3781 (19)	C16—C21	1.379 (2)
N2—C1	1.3998 (18)	C16—C17	1.383 (2)
N2—H2	0.9905	C17—C18	1.378 (2)
N3—C15	1.3327 (19)	C17—H17	0.9300
N3—C16	1.4246 (19)	C18—C19	1.372 (3)
N3—H3	0.9761	C18—H18	0.9300
N4—C22	1.3767 (19)	C19—C20	1.364 (3)
N4—C15	1.4004 (19)	C20—C21	1.384 (2)
N4—H4	0.9287	C20—H20	0.9300
C2—C7	1.376 (2)	C21—H21	0.9300
C2—C3	1.388 (2)	C22—C23	1.488 (2)
C3—C4	1.386 (2)	C23—C24	1.383 (2)
C3—H3A	0.9300	C23—C28	1.396 (2)
C4—C5	1.362 (2)	C24—C25	1.390 (3)
C4—H4A	0.9300	C24—H24	0.9300
C5—C6	1.362 (2)	C25—C26	1.374 (3)
C6—C7	1.386 (2)	C25—H25	0.9300
C6—H6	0.9300	C26—C27	1.372 (3)
C7—H7	0.9300	C26—H26	0.9300
C8—C9	1.4904 (19)	C27—C28	1.382 (2)
C9—C14	1.388 (2)	C27—H27	0.9300
C9—C10	1.389 (2)	C28—H28	0.9300
			
C1—N1—C2	127.39 (13)	C12—C13—C14	120.38 (15)
C1—N1—H1	117.5 (14)	C12—C13—H13	119.8
C2—N1—H1	114.6 (14)	C14—C13—H13	119.8
C8—N2—C1	127.90 (12)	C13—C14—C9	119.97 (15)
C8—N2—H2	118.4	C13—C14—H14	120.0
C1—N2—H2	113.6	C9—C14—H14	120.0
C15—N3—C16	126.08 (12)	N3—C15—N4	115.58 (13)
C15—N3—H3	114.0	N3—C15—S2	126.05 (11)
C16—N3—H3	119.4	N4—C15—S2	118.34 (11)
C22—N4—C15	127.94 (13)	C21—C16—C17	120.04 (15)
C22—N4—H4	116.5	C21—C16—N3	118.07 (14)
C15—N4—H4	115.5	C17—C16—N3	121.74 (14)
N1—C1—N2	115.29 (13)	C18—C17—C16	119.95 (16)
N1—C1—S1	126.60 (11)	C18—C17—H17	120.0
N2—C1—S1	118.10 (11)	C16—C17—H17	120.0
C7—C2—C3	120.12 (14)	C19—C18—C17	118.83 (17)
C7—C2—N1	117.19 (13)	C19—C18—H18	120.6
C3—C2—N1	122.63 (14)	C17—C18—H18	120.6
C4—C3—C2	119.32 (15)	F2—C19—C20	118.72 (18)
C4—C3—H3A	120.3	F2—C19—C18	118.92 (18)
C2—C3—H3A	120.3	C20—C19—C18	122.36 (16)
C5—C4—C3	119.02 (15)	C19—C20—C21	118.61 (17)
C5—C4—H4A	120.5	C19—C20—H20	120.7
C3—C4—H4A	120.5	C21—C20—H20	120.7
F1—C5—C6	118.81 (16)	C16—C21—C20	120.20 (17)
F1—C5—C4	118.31 (15)	C16—C21—H21	119.9
C6—C5—C4	122.88 (15)	C20—C21—H21	119.9
C5—C6—C7	118.19 (15)	O2—C22—N4	121.91 (14)
C5—C6—H6	120.9	O2—C22—C23	121.47 (14)
C7—C6—H6	120.9	N4—C22—C23	116.62 (13)
C2—C7—C6	120.45 (14)	C24—C23—C28	119.36 (15)
C2—C7—H7	119.8	C24—C23—C22	123.87 (14)
C6—C7—H7	119.8	C28—C23—C22	116.76 (14)
O1—C8—N2	123.06 (13)	C23—C24—C25	119.67 (17)
O1—C8—C9	121.47 (13)	C23—C24—H24	120.2
N2—C8—C9	115.47 (13)	C25—C24—H24	120.2
C14—C9—C10	119.75 (13)	C26—C25—C24	120.52 (19)
C14—C9—C8	117.40 (13)	C26—C25—H25	119.7
C10—C9—C8	122.81 (13)	C24—C25—H25	119.7
C11—C10—C9	119.64 (14)	C27—C26—C25	120.09 (18)
C11—C10—H10	120.2	C27—C26—H26	120.0
C9—C10—H10	120.2	C25—C26—H26	120.0
C12—C11—C10	120.43 (15)	C26—C27—C28	120.17 (18)
C12—C11—H11	119.8	C26—C27—H27	119.9
C10—C11—H11	119.8	C28—C27—H27	119.9
C13—C12—C11	119.81 (14)	C27—C28—C23	120.16 (18)
C13—C12—H12	120.1	C27—C28—H28	119.9
C11—C12—H12	120.1	C23—C28—H28	119.9
			
C2—N1—C1—N2	−176.44 (14)	C16—N3—C15—N4	−176.80 (14)
C2—N1—C1—S1	3.8 (2)	C16—N3—C15—S2	1.6 (2)
C8—N2—C1—N1	8.3 (2)	C22—N4—C15—N3	11.7 (2)
C8—N2—C1—S1	−171.98 (13)	C22—N4—C15—S2	−166.79 (13)
C1—N1—C2—C7	136.52 (17)	C15—N3—C16—C21	132.89 (17)
C1—N1—C2—C3	−46.1 (2)	C15—N3—C16—C17	−51.5 (2)
C7—C2—C3—C4	−0.8 (3)	C21—C16—C17—C18	−1.3 (2)
N1—C2—C3—C4	−178.15 (15)	N3—C16—C17—C18	−176.91 (14)
C2—C3—C4—C5	0.9 (3)	C16—C17—C18—C19	0.3 (3)
C3—C4—C5—F1	179.79 (15)	C17—C18—C19—F2	−178.97 (16)
C3—C4—C5—C6	−0.5 (3)	C17—C18—C19—C20	0.8 (3)
F1—C5—C6—C7	179.82 (15)	F2—C19—C20—C21	179.03 (16)
C4—C5—C6—C7	0.2 (3)	C18—C19—C20—C21	−0.7 (3)
C3—C2—C7—C6	0.4 (2)	C17—C16—C21—C20	1.4 (2)
N1—C2—C7—C6	177.91 (15)	N3—C16—C21—C20	177.13 (15)
C5—C6—C7—C2	−0.1 (3)	C19—C20—C21—C16	−0.4 (3)
C1—N2—C8—O1	−8.6 (3)	C15—N4—C22—O2	−8.8 (3)
C1—N2—C8—C9	172.24 (14)	C15—N4—C22—C23	170.92 (14)
O1—C8—C9—C14	−29.9 (2)	O2—C22—C23—C24	165.86 (16)
N2—C8—C9—C14	149.29 (15)	N4—C22—C23—C24	−13.8 (2)
O1—C8—C9—C10	147.74 (18)	O2—C22—C23—C28	−12.5 (2)
N2—C8—C9—C10	−33.1 (2)	N4—C22—C23—C28	167.86 (14)
C14—C9—C10—C11	−0.6 (2)	C28—C23—C24—C25	−1.8 (3)
C8—C9—C10—C11	−178.13 (14)	C22—C23—C24—C25	179.94 (16)
C9—C10—C11—C12	−0.5 (2)	C23—C24—C25—C26	0.6 (3)
C10—C11—C12—C13	0.7 (3)	C24—C25—C26—C27	0.8 (3)
C11—C12—C13—C14	0.3 (3)	C25—C26—C27—C28	−1.0 (3)
C12—C13—C14—C9	−1.4 (3)	C26—C27—C28—C23	−0.3 (3)
C10—C9—C14—C13	1.5 (2)	C24—C23—C28—C27	1.6 (3)
C8—C9—C14—C13	179.19 (15)	C22—C23—C28—C27	−179.96 (16)

### Hydrogen-bond geometry (Å, º)

**Table:**

*D*—H···*A*	*D*—H	H···*A*	*D*···*A*	*D*—H···*A*
N2—H2···S2^i^	0.99	2.56	3.5433 (13)	170
N4—H4···S1^i^	0.93	2.74	3.5976 (13)	154
N1—H1···O1	0.86 (2)	1.95 (2)	2.6408 (17)	137.5 (19)
N3—H3···O2	0.98	1.81	2.6307 (17)	139

Symmetry code: (i) −*x*+1, −*y*+2, −*z*+1.
