# Supplementary figures and images for: Crystal structure of 1-benzoyl-3-(4-fluoro­phen­yl)thio­urea
Source: Acta Crystallogr Sect E Struct Rep Online. 2014 Aug 16;70(Pt 9):o1023–4. doi: 10.1107/S1600536814018376 (PMC4186107; doi:10.1107/S1600536814018376)

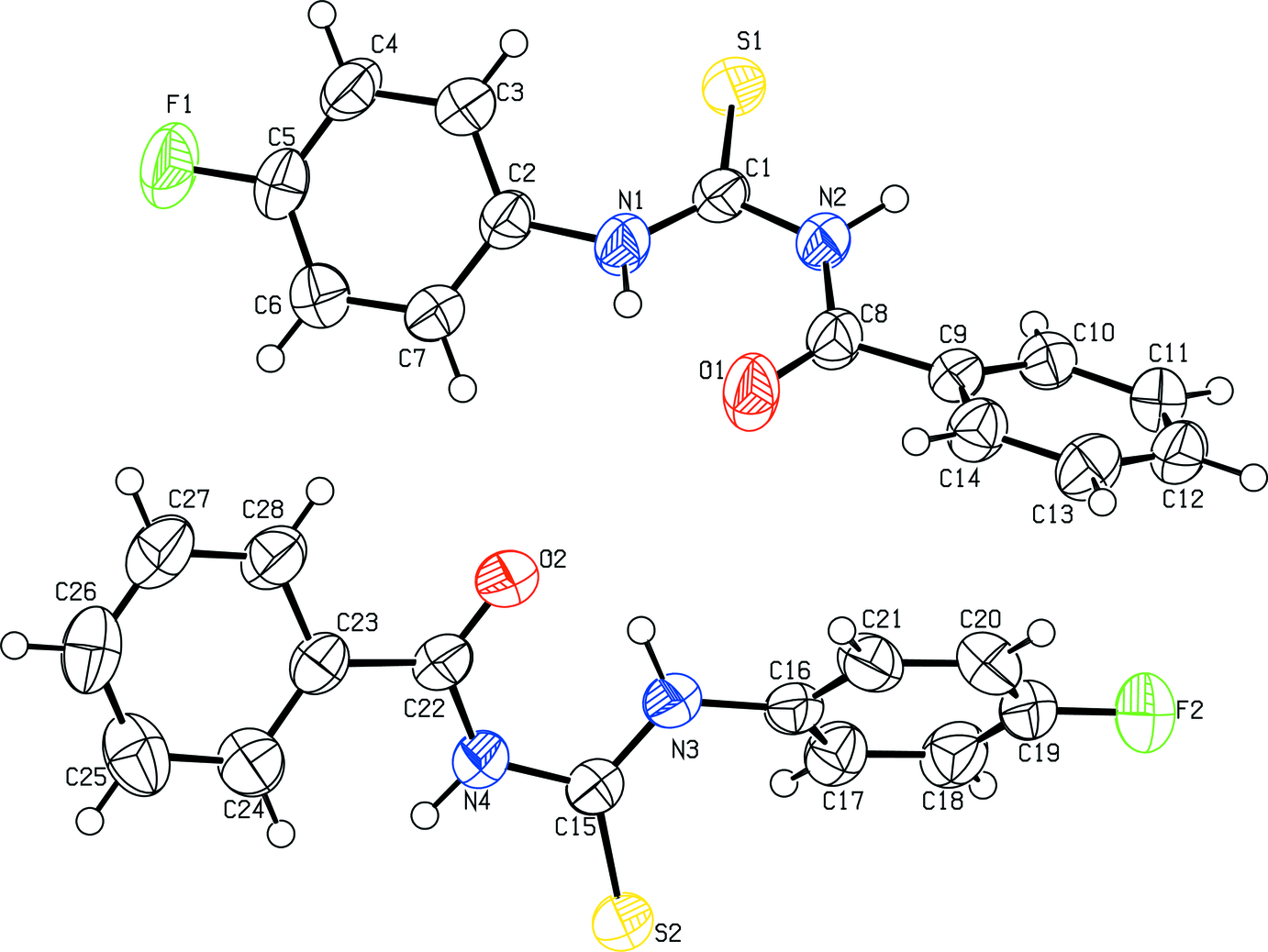

Supplement: Supplementary file 4 [file e-70-o1023-fig1.tif]

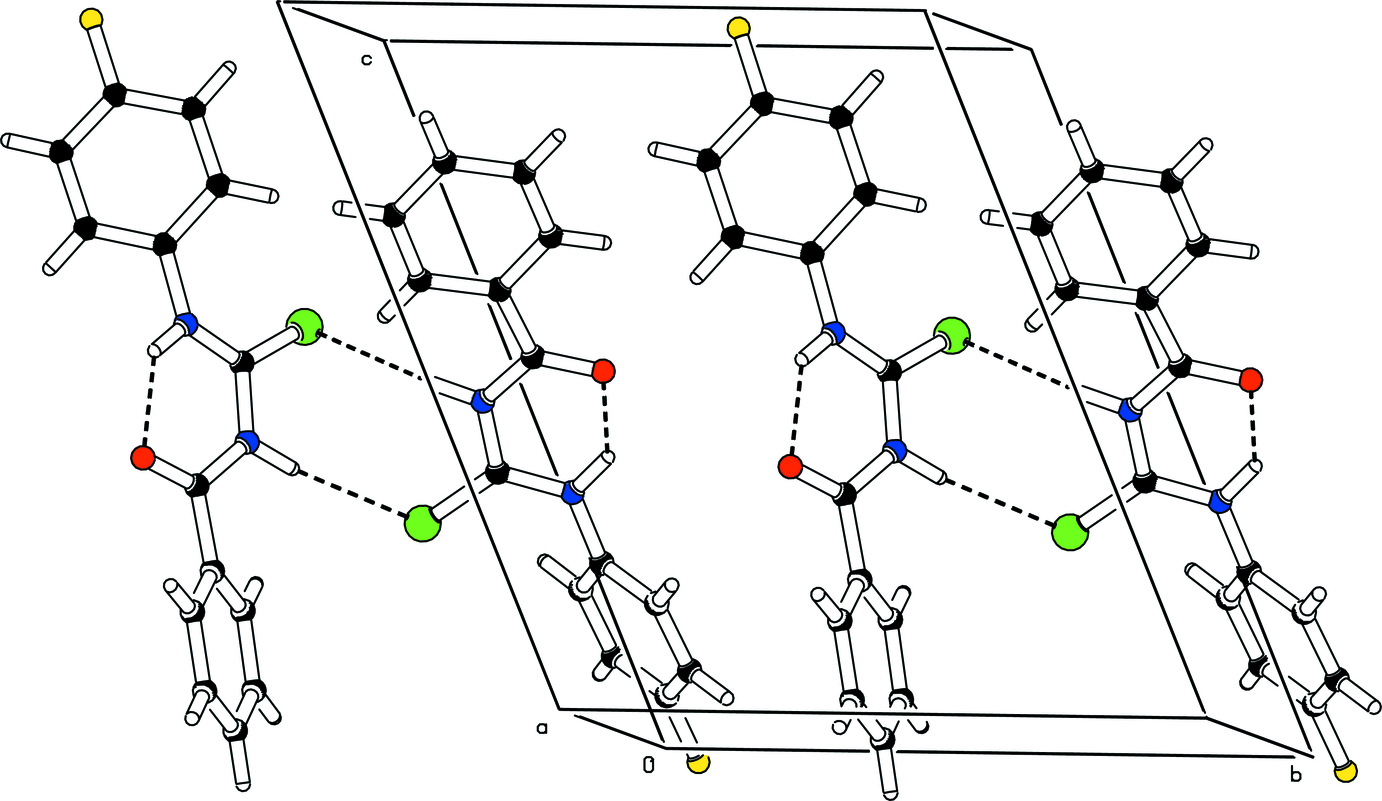

Supplement: Supplementary file 5 [file e-70-o1023-fig2.tif]
